# Author Correction: Back flux during anaerobic oxidation of butane support archaea-mediated alkanogenesis

**DOI:** 10.1038/s41467-024-55458-6

**Published:** 2025-01-07

**Authors:** Song-Can Chen, Sheng Chen, Niculina Musat, Steffen Kümmel, Jiaheng Ji, Marie Braad Lund, Alexis Gilbert, Oliver J. Lechtenfeld, Hans-Hermann Richnow, Florin Musat

**Affiliations:** 1 Division of Microbial Ecology, Center for Microbiology and Environmental Systems Science, University of Vienna, Vienna, Austria; 2 MOE Key Laboratory of Environment Remediation and Ecological Health, College of Environmental and Resource Sciences, Zhejiang University, Hangzhou, China; 3 Research Center for Mathematics, Advanced Institute of Natural Sciences, Beijing Normal University, Zhuhai, China; 4 Department of Biology, Section for Microbiology, Aarhus University, Aarhus, Denmark; 5 Department of Technical Biogeochemistry, Helmholtz Centre for Environmental Research – UFZ, Leipzig, Germany; 6 Department of Earth and Planetary Sciences, Tokyo Institute of Technology, Meguro, Tokyo Japan; 7 Department of Environmental Analytical Chemistry, Helmholtz Centre for Environmental Research – UFZ, Leipzig, Germany; 8 Department of Molecular Biology and Biotechnology, Faculty of Biology and Geology, Babeş-Bolyai University, Cluj-Napoca, Romania

**Keywords:** Archaeal physiology, Carbon cycle, Environmental microbiology

Correction to: *Nature Communications* 10.1038/s41467-024-53932-9, published online 07 November 2024

In this article the affiliation details for Songcan Chen were incorrectly given as “MOE Key Laboratory of Environmental Remediation and Ecological Health, College of Environmental and Resource Sciences, Zhejiang University, Hangzhou 310058, PR China” but should have been ‘MOE Key Laboratory of Environment Remediation and Ecological Health, College of Environmental and Resource Sciences, Zhejiang University, Hangzhou 310058, PR China’. Second, figure 2 has been corrected to make panel labelling consistent with the other figures in the article. The original article has been corrected.

