# Supplementary material for: Back flux during anaerobic oxidation of butane supports archaea-mediated alkanogenesis
Source: Nat Commun. 2024 Nov 7;15:9628. doi: 10.1038/s41467-024-53932-9 (PMC11543930; doi:10.1038/s41467-024-53932-9)
Supplement: Supplementary file 1 — Supplementary Information [file 41467_2024_53932_MOESM1_ESM.pdf]

Supplementary Information

Back flux during anaerobic oxidation of butane support archaea-mediated alkanogenesis

Song-Can Chen[1,2], Sheng Chen[3], Niculina Musat[4], Steffen Kümmel[5], Jiaheng Ji[5], Marie Braad Lund[4], Alexis Gilbert[6], Oliver J. Lechtenfeld[7], Hans-Hermann Richnow[5], Florin Musat[4,8]

[1] Division of Microbial Ecology, Center for Microbiology and Environmental Systems Science, University of Vienna, Vienna, Austria. [2]MOE Key Laboratory of Environmental Remediation and Ecological Health, College of Environmental and Resource Sciences, Zhejiang University, Hangzhou 310058, China. [3] Research Center for Mathematics, Advanced Institute of Natural Sciences, Beijing Normal University; Zhuhai 519087, China. [4] Department of Biology, Section for Microbiology, Aarhus University, Aarhus, Denmark. [5] Department of Technical Biogeochemistry, Helmholtz Centre for Environmental Research – UFZ; Leipzig, Germany. [6] Department of Earth and Planetary Sciences, Tokyo Institute of Technology; Meguro, 152-8551 Tokyo, Japan. [7] Department of Environmental Analytical Chemistry, Helmholtz Centre for Environmental Research – UFZ; Leipzig, Germany. [8] Department of Molecular Biology and Biotechnology, Faculty of Biology and Geology, Babeş-Bolyai University; Cluj-Napoca, Romania

Correspondence to FM: florin.musat@bio.au.dk

Table of Contents

Supplementary Notes 3
Supplementary Note 1 3
Supplementary Note 2 3
Supplementary Note 3 4
Supplementary Note 4 5
Supplementary Note 5 6
Supplementary Note 6 6
Supplementary Note 7 6
Supplementary Note 8 7
Supplementary Note 9 8

Supplementary Figures and Tables 9
Supplementary Figure 1 9
Supplementary Figure 2 10
Supplementary Figure 3 11
Supplementary Figure 4 12
Supplementary Figure 5 13
Supplementary Figure 6 14
Supplementary Figure 7 15
Supplementary Figure 8 16
Supplementary Figure 9 17
Supplementary Figure 10 18
Supplementary Table 1 19
Supplementary Table 2 20
Supplementary Table 3 21
Supplementary Table 4 22
Supplementary Table 5 23
Supplementary Table 6 24
Supplementary Table 7 25
Supplementary Table 8 26

Supplementary references 27

Supplementary Notes

Supplementary Note 1

The putative syntrophic intermediate (X) between *Ca.* S. butanivorans and sulfate reducing bacteria (SRB). Attempts to stimulate a reverse AOB reaction using compounds like H_2, hydrogen sulfide, zero-valent sulfur compounds, or reduced AQDS were unsuccessful. In an attempt to identify X, the presumed redox carrier coupling AOB by *Ca*. S. butanivorans to sulfate reduction by *Ca*. Desulfofervidus auxilii HotSeep-1, we revisited the genome of *Ca.* S. butanivorans. Similar to former analyses [1], we identified genetic systems suggesting the potential to produce H_2, acetate/pyruvate, and cytochrome- or pili-based nanowires.

As formerly shown, *Ca.* S. butanivorans encodes a [NiFe] hydrogenase potentially allowing H_2 formation. However, H_2 was excluded as a mediator of electron transfer in physiology assays showing that its formation rates are too slow to reconcile the observed sulfate reduction rate [1].

Ca. S. butanivorans encodes genes for acetyl-CoA synthase (ACS) and pyruvate:ferredoxin oxidoreductase (POR), suggesting a potential to produce or consume acetate or pyruvate (Supplementary Table 2). However, both compounds are excluded as mediators of electron transfer: physiology assays showed the SRB partner is chemolithoautotrophic, lacking the capacity to utilize acetate or pyruvate [2]. This implies that ACS and POR could be potentially involved in anabolic pathways.

These observations leave open the possibility that *Ca*. S. butanivorans interact with SRB via cytochrome- or pili-based direct interspecies electron transfer. This is supported by the observation of nanowire-like networks and the expression of genes for cytochromes/pili formation in the butane-oxidizing consortia [1].

Supplementary Note 2

Comparative genomics to reveal unknown reactions converting butyl-CoM to butyryl-CoA. To search for enzymes potentially mediating the conversion of butyl-CoM to butyryl-CoA, we compared the genomes of nine anaerobic multicarbon alkane-oxidizing archaea (ANKA) versus twelve anaerobic methanotrophic archaea (ANME). This set includes experimentally validated ANKA (e.g., *Syntrophoarchaeum*, *Methanoliparia*, *Ethanoperedens*), and representatives from three major groups of ANME (e.g., ANME-1, ANME-2, and ANME-3; Supplementary Data 1). The hypothesis underlying this comparative analysis is that enzymes involved in alkyl-CoM/acyl-CoA conversion are conserved among ANKA, since ANME oxidizes methyl-CoM without forming CoA intermediates. To examine the distribution pattern of proteins across archaeal species, we clustered all 47082 proteins from the 21 ANKA and ANME genomes into families using the Markov Cluster (MCL) algorithm, yielding 8338 protein families. Among these, 10 proteins families are over-represented in ANKA versus ANME (adjusted $P < 0.05$; Fisher Exact Test; Supplementary Data 3). These protein families include members involved in multicarbon alkane activation (e.g., AcrA and AcrG), beta oxidation (e.g., acyl-CoA dehydrogenase and methylmalonyl-CoA mutase), and vitamin metabolism (e.g., pyridoxine 5'-phosphate oxidase). However, no proteins were identified with predicted capability to convert the alkyl-CoM thioether to the acyl-CoA thioester. This may be due to (1) lack of

conservation of target proteins across ANKA; (2) recruitment of homologous proteins by ANME for alternative purpose; (3) limited number of experimentally validated ANKA genomes that restricts the power of statistical tests; and/or (4) inaccuracy of annotation. To account for the possibility of poor phylogenetic conservation of enzymes, we also compared ACR-encoding Syntrophoarchaeia (*n* = 7) to their MCR-encoding relatives (*n* = 54) from the same class (Supplementary Data 2). The comparison yielded over 500 protein families that are characteristic of ACR-encoding Syntrophoarchaeia (adjusted P < 0.05; Fisher Exact Test; Supplementary Data 4). However, pinpointing the enzymes for alkyl-CoM/acyl-CoA conversion from the predicted candidates would require extensive physiology, metabolomics, and biochemistry assays.

Supplementary Note 3

Temperature dependency of reversibility for the AOB process. The impact of temperature on the reversibility of AOB was assessed by estimating equilibrium constants at two different temperatures. The relationship between equilibrium constants ($K_{eq,1}$ and $K_{eq,2}$) and temperatures (T_1 and T_2) could be approximated by the van't Hoff equation (eq. **S1**), assuming standard enthalpy changes for the AOB process (ΔH^0) is constant over the range of physiologically relevant temperature, e.g., from 25 °C to 50 °C.

$$ln\frac{K_{eq,2}}{K_{eq,1}} = \frac{\Delta H^\circ}{R}(\frac{1}{T_1} - \frac{1}{T_2})$$ eq. **S1**

Where R represents the gas constant (8.314 x 10^{-3} kJ mol^{-1} K^{-1}). The ΔH^0 is the stoichiometric sum of standard enthalpy of formation (ΔH_f^0) for reactants and products of the AOB process (Supplementary Table 5).

$$\Delta H^0 = 4 \times \Delta H_f^0[HCO_3^-] + 3.25 \times \Delta H_f^0[HS^-] + \Delta H_f^0[H_2O] + 0.75 \times \Delta H_f^0[H^+]$$
$$-\Delta H_f^0[C_4H_{10}] - 3.25 \times \Delta H_f^0[SO_4^{2-}]$$
= 4 × (–689.93) + 3.25 × (–20.62) + (–285.83) + 0.75 × 0 – (–74.9) – 3.25 × (–909.3) kJ mol^{-1}
= –82.44 kJ mol^{-1}

eq. **S2**

The ratio of equilibrium constants at T_1 = 298 K ($K_{eq,298K}$) and at T_2 = 323 K ($K_{eq,323K}$) was then calculated,

$$\frac{K_{eq,323K}}{K_{eq,298K}} = exp^{\frac{\Delta H^\circ}{R}(\frac{1}{T_1}-\frac{1}{T_2})} = exp^{\frac{-82.44}{8.314\times10^{-3}}\left(\frac{1}{298}-\frac{1}{323}\right)} = 0.076$$ eq. **S3**

Eq. **S3** indicates a smaller equilibrium constant at 50 °C vs. 25 °C, which points to a more favored rAOB at 50 °C, and thus higher reversibility. The inversely proportional relationship between equilibrium constant and reversibility could be seen from flux force theorem and fundament equations of Gibbs free energy (eq. **S4**), where f_-/f_+ is the backward-to-forward flux ratio, Q is the reaction quotient (the ratio of the

product to reactant activities), R is the gas constant, T is the temperature in Kelvin and K_{eq} is the equilibrium constant (the ratio of product to reactant activities at equilibrium).

$$\frac{f_-}{f_+} = e^{\frac{\Delta G}{RT}} = e^{\frac{\mathrm{RTln}\frac{Q}{K_{eq}}}{RT}} = \frac{Q}{K_{eq}} \qquad \text{eq. } \mathbf{S4}$$

Supplementary Note 4

Bicarbonate/CO_2 equilibrium. To control the amount of ^{13}C added in the labeling experiments, cultures were prepared in Tris-buffered ASW media under a headspace of pure N_2. Bicarbonate was added as specified in the Methods section. Under the experimental conditions, the added bicarbonate (HCO_3^-) can either dissociate into carbonate (CO_3^{2-}), or become protonated to produce aqueous CO_2 (CO_2 (aq)), which can be further partitioned into gas phase (CO_2 (g)). These processes establish chemical equilibrium among different inorganic carbon species in the timeframe of minutes [3]. The quantity of each species can be deduced from stoichiometric equilibrium constants:

$$K_0 = \frac{[CO_2(aq)]}{f_{CO_2}} \qquad K_1 = \frac{[HCO_3^-][H^+]}{[CO_2(aq)]} \qquad K_2 = \frac{[CO_3^{2-}][H^+]}{[HCO_3^-]} \qquad \text{eq } \mathbf{S5}$$

where brackets represent equilibrium concentration (unit: M); f_{CO_2} is the partial pressure of CO_2 in gaseous phase (unit: atm); K_0 is the solubility coefficient of CO_2 in seawater according to Henry's law (unit: M atm^{-1}); K_1 and K_2 are the first and second dissociation constants of carbonic acids.

The sum of all forms of inorganic carbon is equal to the amount of carbon initially added as bicarbonate in a closed system (mass conversation; eq. **S6**).

$$n(CO_2(g)) + [CO_2(aq)]V_l + [HCO_3^-]V_l + [CO_3^{2-}]V_l = [HCO_3^-]_0 V_l \qquad \text{eq } \mathbf{S6}$$

$$n(CO_2(g)) = \frac{V_g \cdot f_{CO_2}}{RT} \qquad \text{eq } \mathbf{S7}$$

where $n(CO_2(g))$ is the amount of carbon in gaseous CO_2 phase (unit: mol), which can be calculated from the partial pressure of CO_2 (f_{CO_2}) using the ideal gas equation (eq. **S7**); $[HCO_3^-]_0$ is the initial concentration of bicarbonate added (unit: M); V_l and V_g are the volume of medium and headspace in the closed system (unit: l), respectively; R is the gas constant; T is the temperature (unit: K).

Combining eq. **S5-S7,** the equilibrium concentrations of the four inorganic carbon species can be expressed as a function of $[HCO_3^-]_0$ and pH (eq. **S8-S11**).

$$[HCO_3^-] = \frac{[HCO_3^-]_0}{\left(1+\frac{V_g/V_l}{K_0 RT}\right)10^{pK_1-pH}+10^{pH-pK_2}+1} \qquad \text{eq } \mathbf{S8}$$

$$[CO_3^{2-}] = 10^{pH-pK_2}[HCO_3^-] \qquad \text{eq } \mathbf{S9}$$

$$[CO_2(aq)] = 10^{pK_1 - pH}[HCO_3^-]$$ eq **S10**

$$f_{CO_2} = \frac{[CO_2(aq)]}{K_0}$$ eq **S11**

where pK and pH are the negative common logarithm of equilibrium constants and concentration of proton ([H^+]), respectively.

To calculate carbonate speciation under conditions of labelling experiment, we assume Tris-buffered ASW media has a salinity S = 30 g l^{-1} and pH = 7.0 at T = 323 K (50 °C). Addition of 18 mM bicarbonate leads to equilibrium concentration of $[HCO_3^-]$ = 15.4 mM, $[CO_3^{2-}]$ = 0.44 mM, $[CO_2(aq)]$ = 0.68 mM, and f_{CO_2} = 4% atm. The equilibrium constants [4], gas constant, and volumes used for the calculation are K_0 = 0.01698 M atm^{-1}, pK_1 = 5.64, pK_2 = 8.54, R = 0.08205 l atm K^{-1} mol^{-1}, V_l = 60 ml, and V_g = 60 ml.

Supplementary Note 5

Dilution of ^{13}C in the CO_2 pool during net AOB. During the experiment the initially added ^{13}C label is diluted by CO_2 formed through the oxidation of unlabeled butane (natural abundance of ^{13}C). Thus, the culture supplied with 98% ^{13}C-dissolved inorganic carbon (DIC) will reach 42% ^{13}C-DIC at the end of the experiment (Figure 3).

Supplementary Note 6

Determination of isotopic fractionation factor associated with AOB. The isotopic fractionation is determined using following equation:

$$\ln\left(\frac{\delta_t+1000}{\delta_0+1000}\right) = (\alpha_+ - 1)\ln\left(\frac{C_t}{C_0}\right)$$ eq **S12**

where C_t and C_0 are the concentration, and δ_t and δ_0 are the δ^{13}C signature of the butane at a certain time (*t*) and at the beginning of incubation experiment (0), respectively. The data used for the estimation was from the AOB incubation experiments with $^{13}CO_2$ at natural abundance. The isotopic fractionation factor α_+ was obtained by plotting $\ln(C_t/C_0)$ versus $\ln[(\delta_t+1)/(\delta_0+1)]$ and using the slope of the linear regression line (Supplementary Figure 5). Its uncertainty is given as 95% confidential interval derived from regression analysis [5]. The isotopic fractionation ε_+ was calculated according to $\varepsilon_+ = 1000(\alpha_+ - 1)$ [5].

Supplementary Note 7

Determination of the Gibbs free energy of AOB with sulfate. The net AOB reaction catalyzed by the Butane50 culture proceeds according to:

$C_4H_{10} + 3.25\ SO_4^{2-} \rightarrow 4\ HCO_3^- + 3.25\ HS^- + 0.75\ H^+ + H_2O$

The actual Gibbs free energy of the net AOB reaction was calculated according to:

$$\Delta G = \Delta G^{0\prime} + RTln\frac{\{HCO_3^-\}^4\{HS^-\}^{3.25}\{H^+\}^{0.75}\{H_2O\}}{\{C_4H_{10}\}\{SO_4^{2-}\}^{3.25}} \qquad \text{eq } \mathbf{S13}$$

where the curly brackets denote activities of substrates and products of AOB; $\Delta G^{0\prime}$ stands for the standard energy of reaction (pH = 7); *R* is the gas constant and *T* is the temperature of the system. $\Delta G^{0\prime}$ was calculated from standard Gibbs energy of formation for reactants and products (ΔG_f^0 in Supplementary Table 5). The activities of the reaction components in seawater were related to their concentration using activity coefficients (γ in Supplementary Table 5). The $\Delta G^{0\prime}$ for anaerobic oxidation of gasous alkanes were calculated similarly (Supplementary Table 4).

Supplementary Note 8

Calculation of the free energy for the release of butane from butyl-CoM under standard conditions. The standard free energy change of butane formation from butyl-CoM ($\Delta G_1^{0\prime}$) was estimated from the last step of methanogenesis (methyl-CoM reduction) using the group contribution method [6].

The conversion of butyl-CoM to butane is represented by the reaction.

C_4H_9-S-CoM + HS-CoB → C_4H_{10} (g) + CoM-S-S-CoB $\qquad \Delta G_1^{0\prime}$

$\Delta G_1^{0\prime}$ is the stoichiometric sum of the formation energy of reactants ($\Delta_f G^{0\prime}$).

$\Delta G_1^{0\prime} = \Delta_f G^{0\prime}[C_4H_{10}\text{ (g)}] + \Delta_f G^{0\prime}[\text{CoM-S-S-CoB}] - \Delta_f G^{0\prime}[\text{HS-CoB}] - \Delta_f G^{0\prime}[C_4H_9\text{-S-CoM}]$ eq **S14**

The free energy change of the last step of methanogenesis ($\Delta G_2^{0\prime}$) is –30 kJ mol^{-1} [7].

CH_3-S-CoM + HS-CoB → CH_4 (g) + CoM-S-S-CoB $\qquad \Delta G_2^{0\prime} = -30 \pm 10$ kJ mol^{-1}

Similarly, $\Delta G_2^{0\prime}$ is the stoichiometric sum of reactant formation energy (eq. **S15**).

$\Delta G_2^{0\prime} = \Delta_f G^{0\prime}[CH_4\text{ (g)}] + \Delta_f G^{0\prime}[\text{CoM-S-S-CoB}] - \Delta_f G^{0\prime}[\text{HS-CoB}] - \Delta_f G^{0\prime}[CH_3\text{-S-CoM}]$ eq **S15**

The Gibbs free energy of formation for CH_4 (g) and C_4H_{10} (g) are –50.7 kJ mol^{-1} and –17.2 kJ mol^{-1}, respectively (eq. **S16**-**17**) [8].

$\Delta_f G^{0\prime}[CH_4\text{ (g)}] = -50.7$ kJ mol^{-1} eq **S16**
$\Delta_f G^{0\prime}[C_4H_{10}\text{ (g)}] = -17.2$ kJ mol^{-1} eq **S17**

Decomposition of compound into structural groups revealed that butyl-CoM and methyl-CoM differed by three groups of secondary carbon (-CH_2-), which was estimated to contribute 6.8 ± 0.4 kJ mol^{-1} to the

standard energy of formation ($\Delta_{gr}G^{0\prime}$[-CH_2-]) [6]. Using group contribution assumption, the difference between formation energy of butyl-CoM and methyl-CoM can be estimated from $\Delta_{gr}G^{0\prime}$[-CH_2-] (eq. **S18**).

$\Delta_f G^{0\prime}[C_4H_9\text{-S-CoM}] - \Delta_f G^{0\prime}[CH_3\text{-S-CoM}] = 3 \times \Delta_{gr}G^{0\prime}[\text{-}CH_2\text{-}] = 20.4 \pm 1.2$ kJ mol^{-1} eq **S18**

The difference of $\Delta G_1^{0\prime}$ and $\Delta G_2^{0\prime}$ are calculated using eq. **S14**-**S18**:

$\Delta G_1^{0\prime} - \Delta G_2^{0\prime} = (\Delta_f G^{0\prime}[C_4H_{10}\ (g)] - \Delta_f G^{0\prime}[CH_4\ (g)]) - (\Delta_f G^{0\prime}[C_4H_9\text{-S-CoM}] - \Delta_f G^{0\prime}[CH_3\text{-S-CoM}]) = 13.1 \pm 1.2$ kJ mol^{-1}

$\Delta G_1^{0\prime}$ is yielded from known value of $\Delta G_2^{0\prime}$ and the difference between $\Delta G_1^{0\prime}$ and $\Delta G_2^{0\prime}$:

$\Delta G_1^{0\prime} = -16.9 \pm 10$ kJ mol^{-1}

Similarly, the free energy change associated with propane and ethane formation from their CoM precursors were estimated to be –19.0 ± 10 kJ mol^{-1}and –18.5 ± 10 kJ mol^{-1}, respectively.

Supplementary Note 9

Phylogenetic analysis of FrhB/FdhB in *Ca*. S. butanivorans. A search for FrhB/FdhB in *Ca*. S. butanivorans genome yielded seven homologs, all containing the C-terminus domain of coenzyme F420 hydrogenase / dehydrogenase (PF04432). Phylogenetic analysis revealed six of them fall within distinct monophyletic clades spanned by anaerobic methanotrophic (ANME) and methanogenic archaea (Supplementary Figure 10). Notably, the homolog SBU_000300, placed within the FrhB4 clade, is located adjacent to genes for heterodisulfide reductase complexes (HdrABC). This gene arrangement pattern supports an enzymatic interaction between HdrABC complex and FrhB, potentially facilitating confurcation of ferredoxin and CoM-SH/CoB-SH electrons to $F_{420}H_2$ (Reaction 2 in Table 1). The remaining FrhB/FdhB homologs were found in gene clusters containing genes for F_{420}:quinone oxidoreductase (Fqo), glutamate synthase (GltS), pyruvate:ferredoxin oxidoreductase (Por), or HdrA (Supplementary Figure 10). Similar gene context has been observed in many ANME-1 genomes, suggesting a link of F_{420} redox reactions with a range of electron transport capacities, such as energy transduction, amino acid metabolism, redox transformation of pyruvate, or flavin-based electron bifurcation/confurcation.

Supplementary Figures and Tables

Supplementary Figure 1

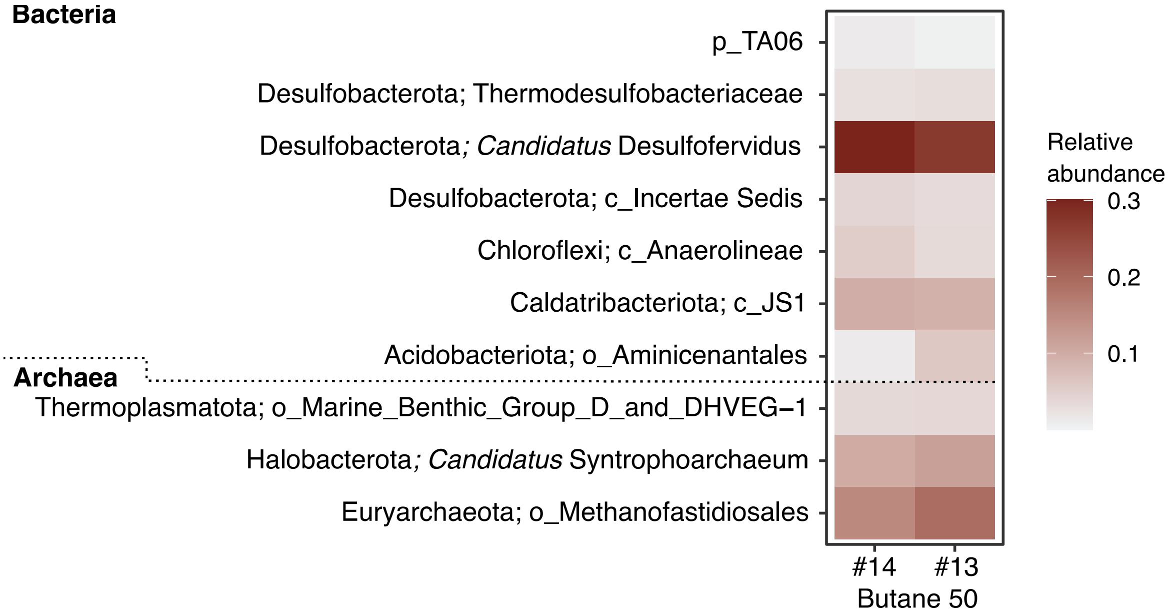

Supplementary Figure 1| Relative abundance of genera comprising over 1% of the total microbiome in two independent Butane50 cultures (#13 and #14), based on amplicon sequencing of the V3-V4 region of the 16S rRNA gene. Some taxa could only be classified to the level of class or order (prefixed with 'c_' or 'o_', respectively). Amplicon sequencing showed a strong bias against *Ca*. Syntrophoarchaeum, which prompted us to characterize the microbial community by catalyzed reporter deposition – fluorescence *in situ* hybridization (CARD-FISH) and direct cell counting. Source data are provided as a Source Data file.

Supplementary Figure 2

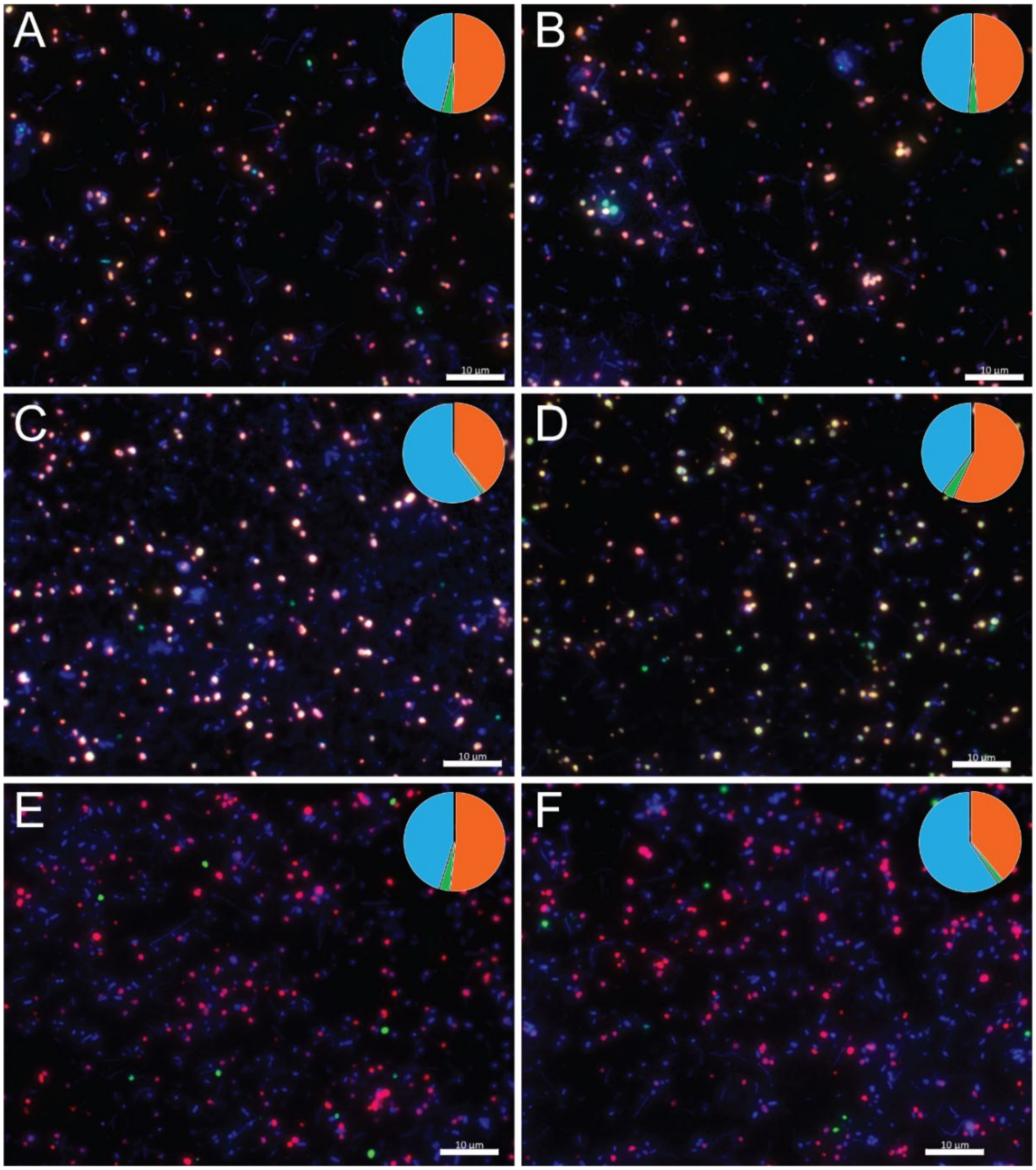

Supplementary Figure 2 | Additional microscopy images of the Butane50 culture showing *Ca*. S. butanivorans as the dominant archaeon. A-D. Double hybridization of dispersed aggregates with oligonucleotide probes specific for Archaea (green), and for *Ca*. S. butanivorans (orange; *Ca*. S. butanivorans cells were hybridized with both probes). **E-F.** Double hybridization of dispersed aggregates with probes specific for *Ca*. S. butanivorans (magenta) and *Ca*. S. caldarius (green). Insets in each image show the abundance of *Ca*. S. butanivorans (orange, all panels), other Archaea (green, panels A-D), *Ca*. S. caldarius (green, panels E-F), and Bacteria (blue, all panels). Panels A-B, C-D, and E-F are representative of independent cultures (*n* = 3), respectively, collected at different time points during growth. Images are representative of *n* > 60 collected images. *Ca*. S. butanivorans and *Ca*. S. caldarius abundance is based on *n* > 5000 counted cells. Scale bars = 10 µm, applicable to all panels.

Supplementary Figure 3

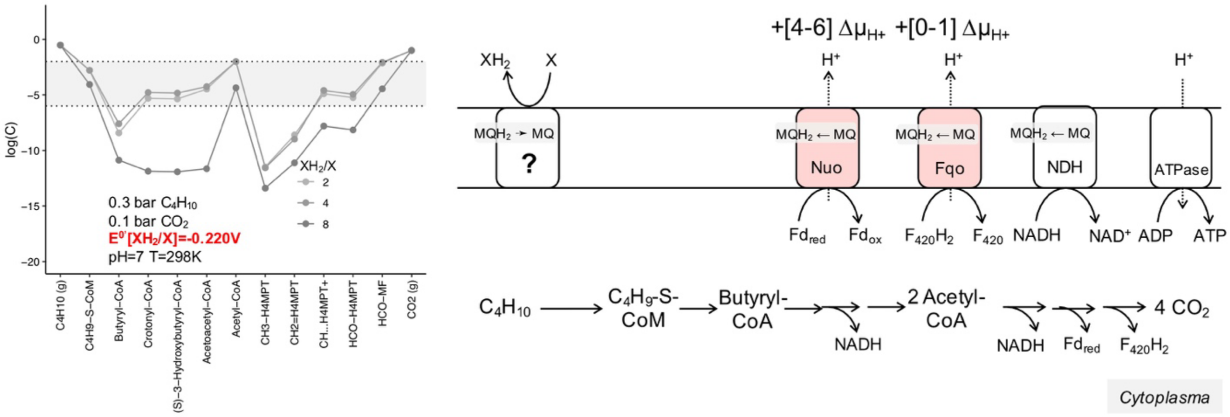

Supplementary Figure 3 | Modelled quasi-equilibrium concentrations (mol L^{-1}) of all AOB pathway metabolites, considering $F_{420}H_2$ and Fd_{red} oxidation as the only energy recovery sites. No energy dissipation or energy investment were assumed in other individual reactions. The concentrations were calculated under different XH_2/X ratios (2, 4, and 8), returning overall AOB Gibbs free energies (ΔG_{cat}) of −147.23, −124.91, and −102.58, kJ mol^{-1} butane. The corresponding Gibbs free energy change for sulfate reduction was −13.56, −20.43, and −27.30 kJ mol^{-1} sulfate, assuming 5 mM sulfide and 28 mM sulfate; $\Delta\mu_{H+}$ denotes the free energy required to translocate one proton across the membrane (−20 kJ mol^{-1}). The standard redox potential ($E^{0\prime}$) of XH_2/X was −0.220 V, corresponding to the average redox potential of sulfate reduction.

Supplementary Figure 4

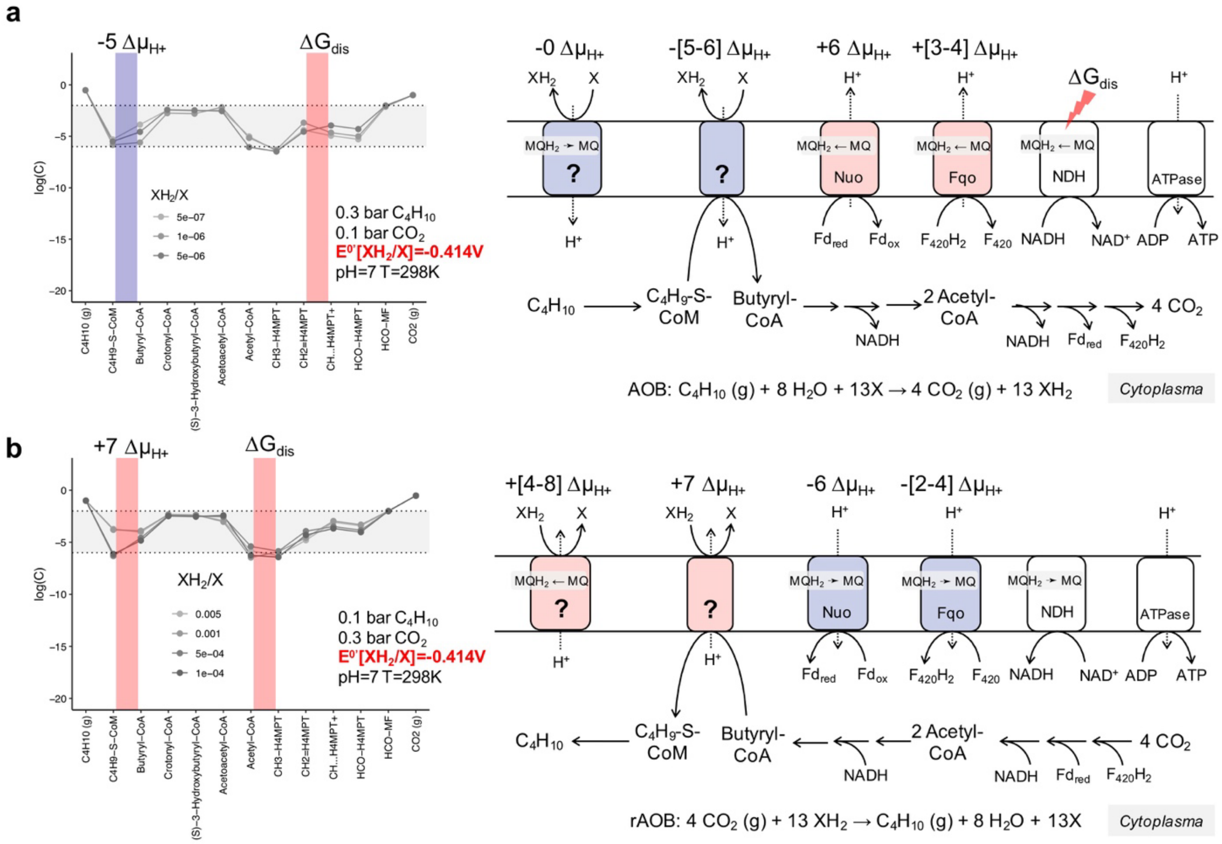

Supplementary Figure 4 | Quasi-equilibrium concentration (mol L^{-1}) of AOB and rAOB pathway with the standard redox potential of X set at −0.414V. a. The AOB model considers energy recovery at $F_{420}H_2$ and Fd_{red} oxidation, energy investment at butyl-CoM/butyryl-CoA conversion and electron transport between MQ and X, and energy dissipation (ΔG_{dis}) at the oxidation of methylene-tetrahydromethanopterin and NADH. The metabolite concentrations were calculated under different XH_2/X ratios, returning overall AOB Gibbs free energy changes of (ΔG_{cat}) of −150.11, −127.79, and −75.95 kJ mol^{-1} butane, respectively. **b**. The rAOB model considers a switch in energy recovery and energy investment, with energy dissipation at acetyl-CoA synthesis. The metabolite concentrations were calculated under different XH_2/X ratios, with overall Gibbs free energy change for rAOB reaction (ΔG_{cat}) of −34.15, −85.98, −108.31, and −160.15, kJ mol^{-1} butane, respectively. $\Delta\mu_{H+}$ denote the free energy required to translocate one proton across the membrane (−20 kJ mol^{-1}). The standard redox potential ($E^{0\prime}$) of XH_2/X was set to −0.414 V, corresponding to the redox potential of H_2/H^+ redox pair.

Supplementary Figure 5

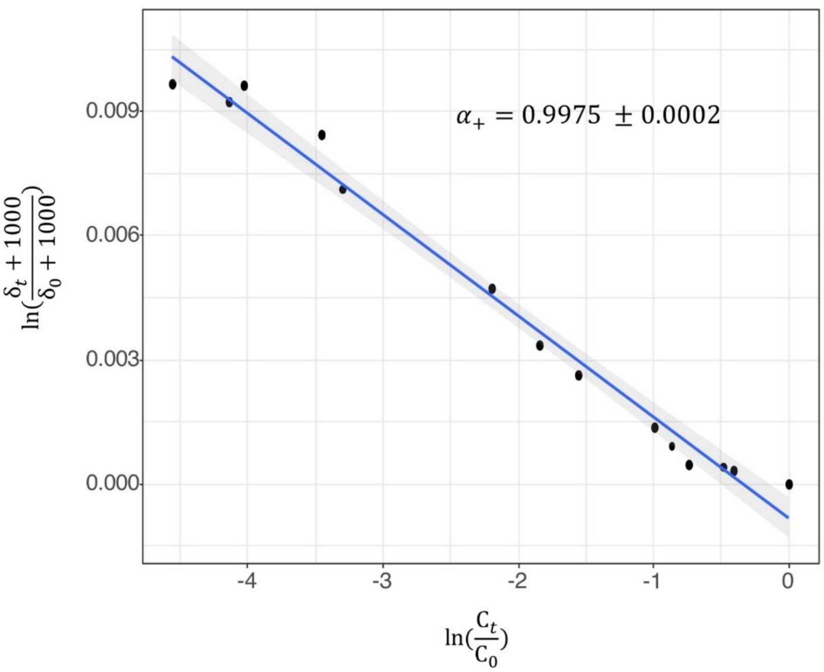

Supplementary Figure 5| Isotopic fractionation factor (α_+) associated with AOB determined by double logarithmic plot. The circles are the experimental data from the incubation with CO_2 at natural ^{13}C abundance. The linear regression line is shown in blue. The envelope (gray) marks the 95% confidence interval of predictions by linear regression. The estimate value of α_+ together with its 95% confidence interval is shown.

Supplementary Figure 6

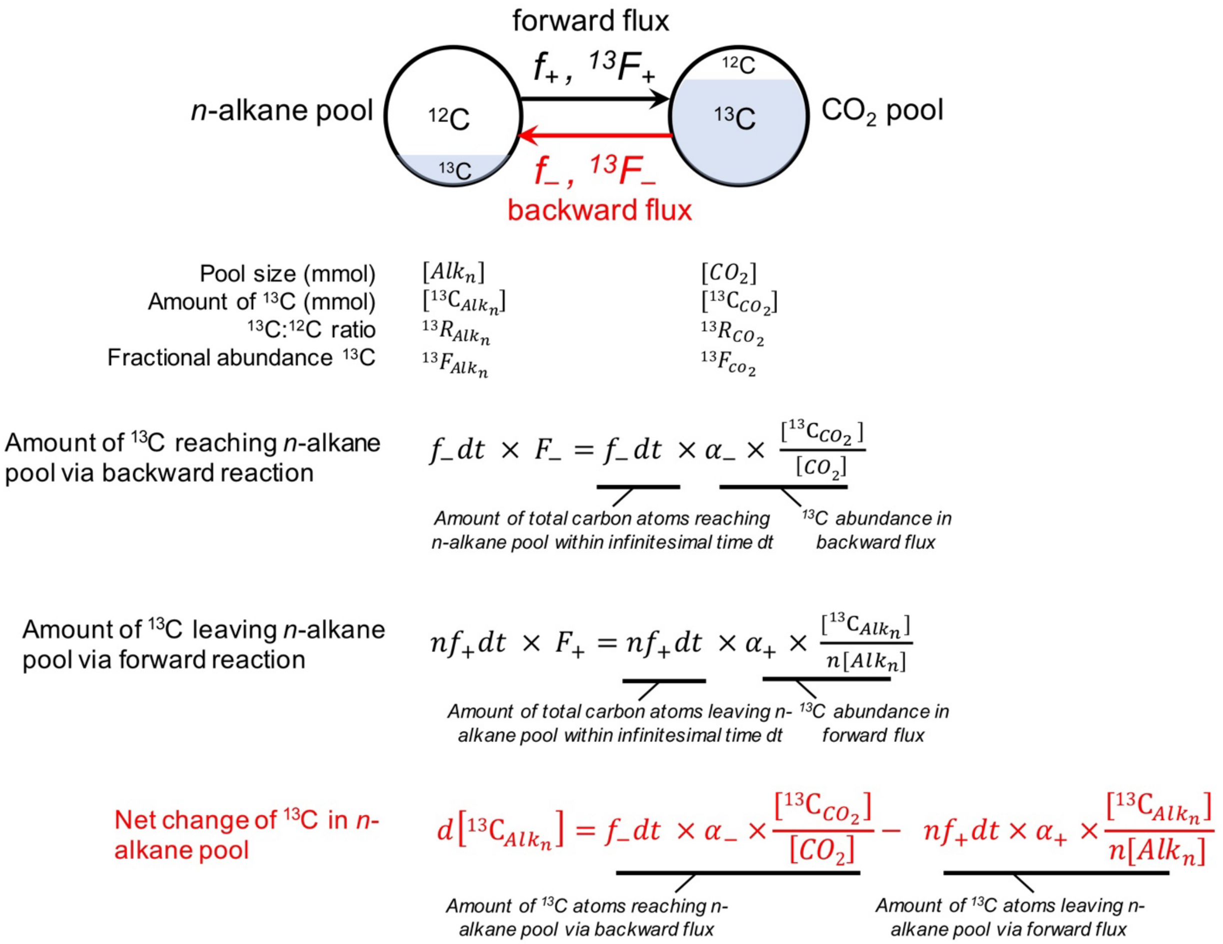

Supplementary Figure 6 | Graphic illustration of the isotope mass balance model. The model considers anaerobic oxidation of *n*-alkane as a reversible process. Both forward and backward fluxes affect the isotope composition of *n*-alkane: the back flux brings ^{13}C from the CO_2 pool, whereas the forward flux takes away ^{13}C from the *n*-alkane pool. Within infinitesimal time intervals (dt), the amount of ^{13}C transferred via back flux equals to the total amount of carbon ($f_-\mathrm{d}t$) times fractional abundance of ^{13}C in the flux ($^{13}F_-$). The latter can be further related to the ^{13}C abundance in the CO_2 pool ($^{13}F_{CO_2}$) using the isotopic fractionation factor ($\alpha_- \approx {}^{13}F_-/{}^{13}F_{CO_2}$). The amount of ^{13}C transferred via forward flux can be derived similarly. These two values give rise to the net change of ^{13}C in *n*-alkane with dt (eq. 2 in the main text).

Supplementary Figure 7

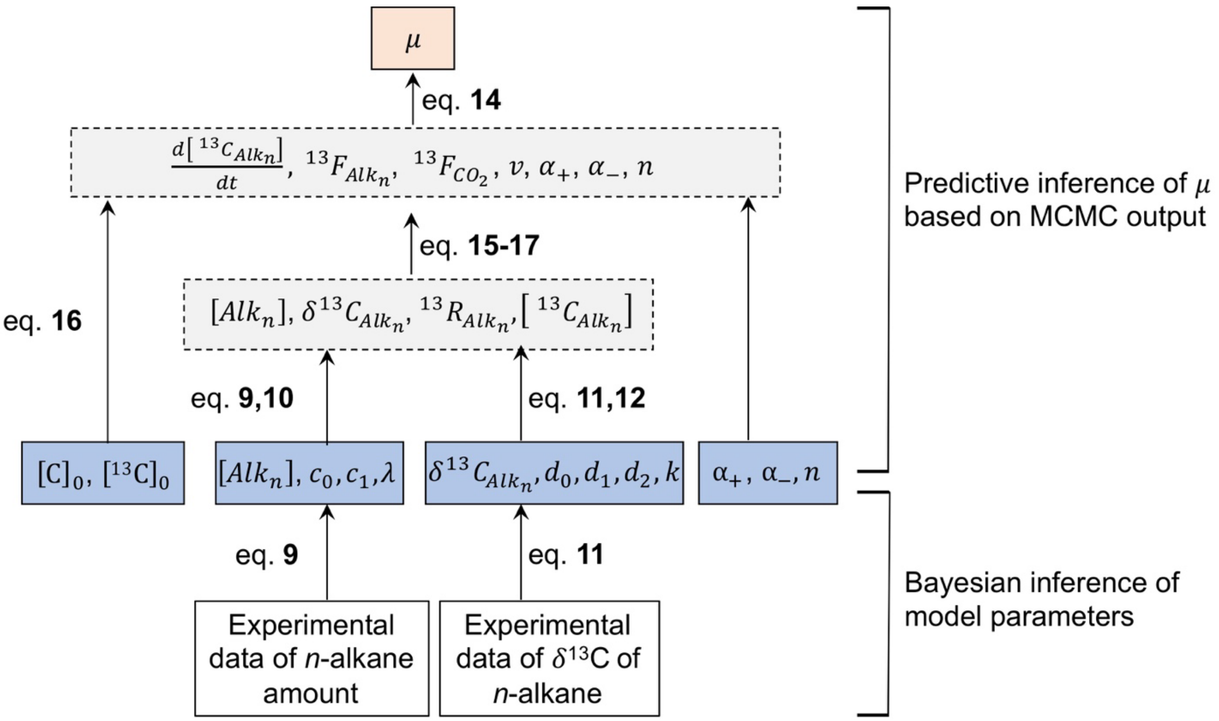

Supplementary Figure 7 | Derivation tree of back flux extent (μ). Derivations tree shows that μ is dependent on (1) initial amounts of carbon and ^{13}C supplemented to the system ($[C]_0$ and $[^{13}C]_0$); (2) parameters in dynamic models (c_0, c_1, λ; d_0, d_1, d_2, k); (3) fractionation factors (α_- and α_+); and (4) number of carbon atoms (n) in the *n*-alkane. Initial conditions are known from experimental setup; parameters in first-order reaction model (c_0, c_1, λ) and logistic model (d_0, d_1, d_2, k) can be inferred from measured data using Bayesian framework; α_+ is obtained from fractionation experiments (Supplementary Figure 5) and α_- is set to 1. The arrows show the relationships between variables and/or constants, with underlying equations indicated beside (see Method). The key variables for the calculation of μ are placed in blue boxes, whereas intermediate variables are in the gray boxes.

Supplementary Figure 8

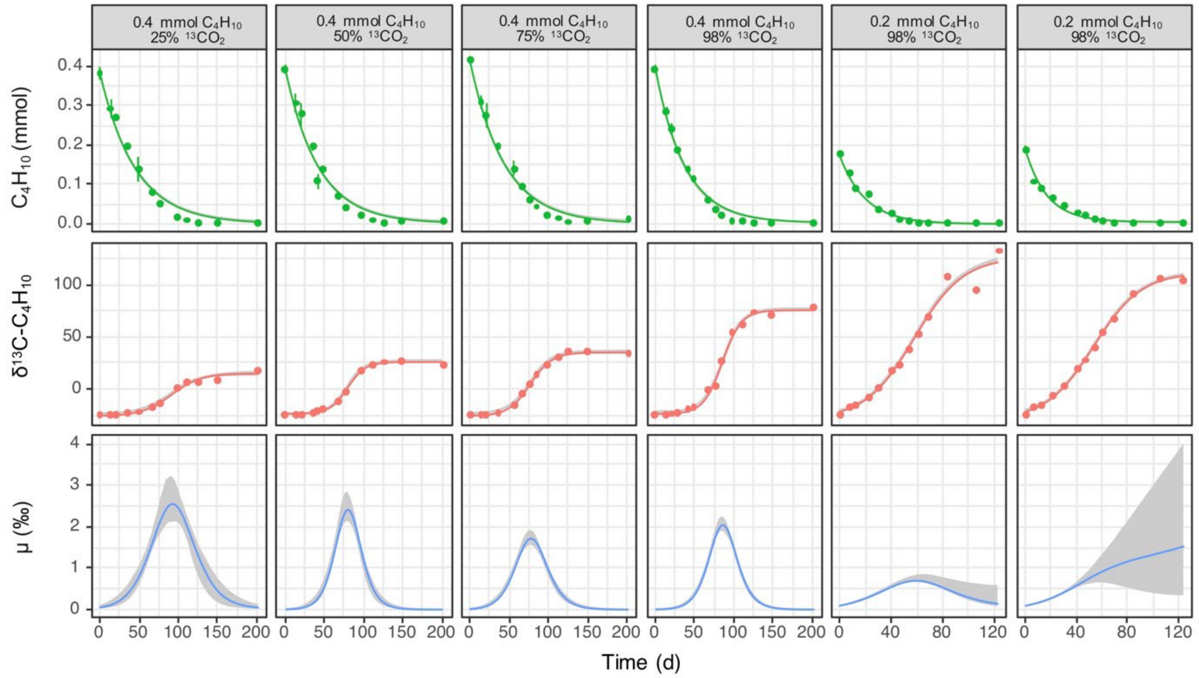

Supplementary Figure 8 | Back flux extent (μ) of AOB inferred from replicate experimental assays. The temporal evolution of butane amount and $\delta^{13}C$ values are shown for comparison. The circles show the experimental data. The error-bars denote the standard deviation among technical replicates (*n*=3). The lines are the posterior mean of the model results. The envelope (gray) marks the 95% credible interval of the model predictions.

Supplementary Figure 9

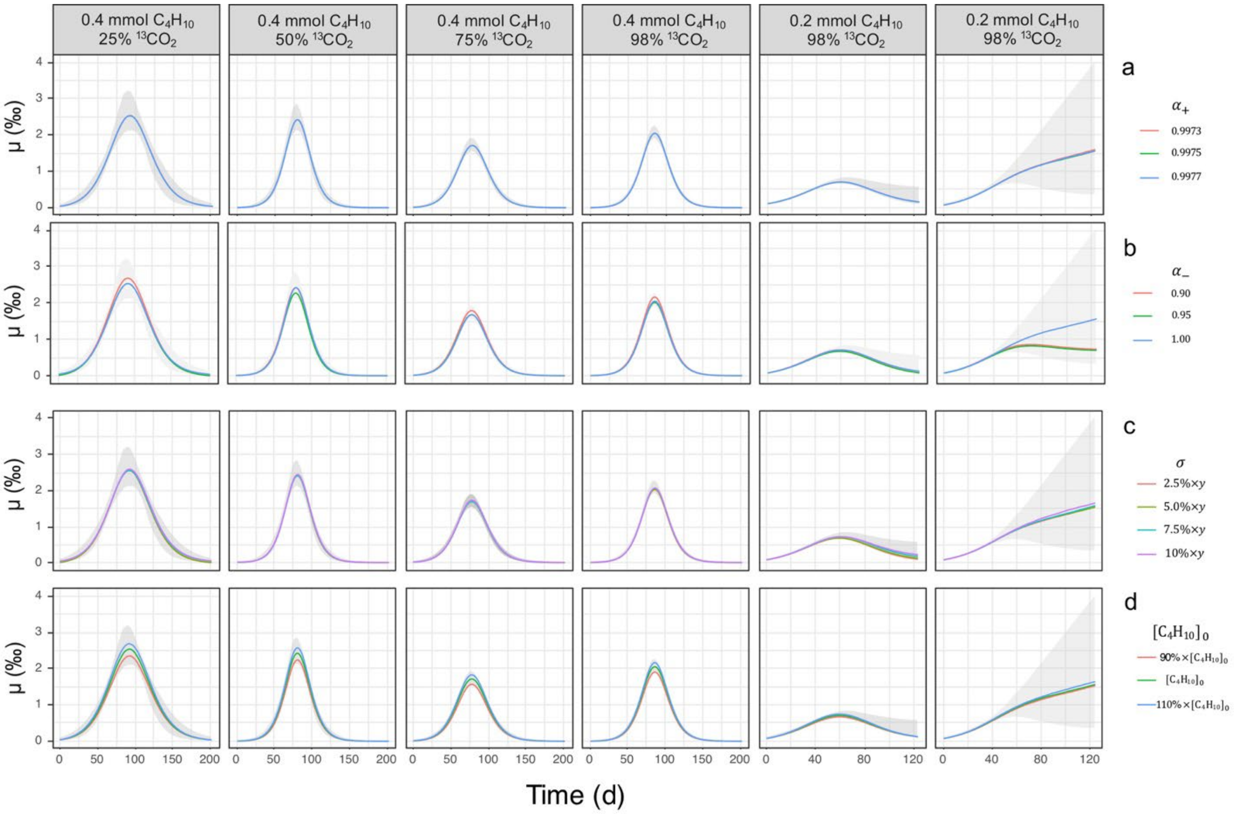

Supplementary Figure 9 | Sensitivity of model results to the fractionation factor α_+ (a), fractionation factor α_- (b), error term σ of the likelihood function (c), and initial concentration of total inorganic carbon $[CO_2]_0$ (d). The simulations were performed using α_+ values ranging from 0.9973 to 0.9977 (default: 0.9975) (**a**); α_- values between 0.90 and 1 (default: 1) (**b**); σ reflecting high (10%), medium (5%; default) or low level (2.5%) of data noise (**c**); and target $[CO_2]_0$ values plus 10% analytical error (**d**). The envelope (gray) marks the 95% credible interval of the model predictions using default model parameters.

Supplementary Figure 10

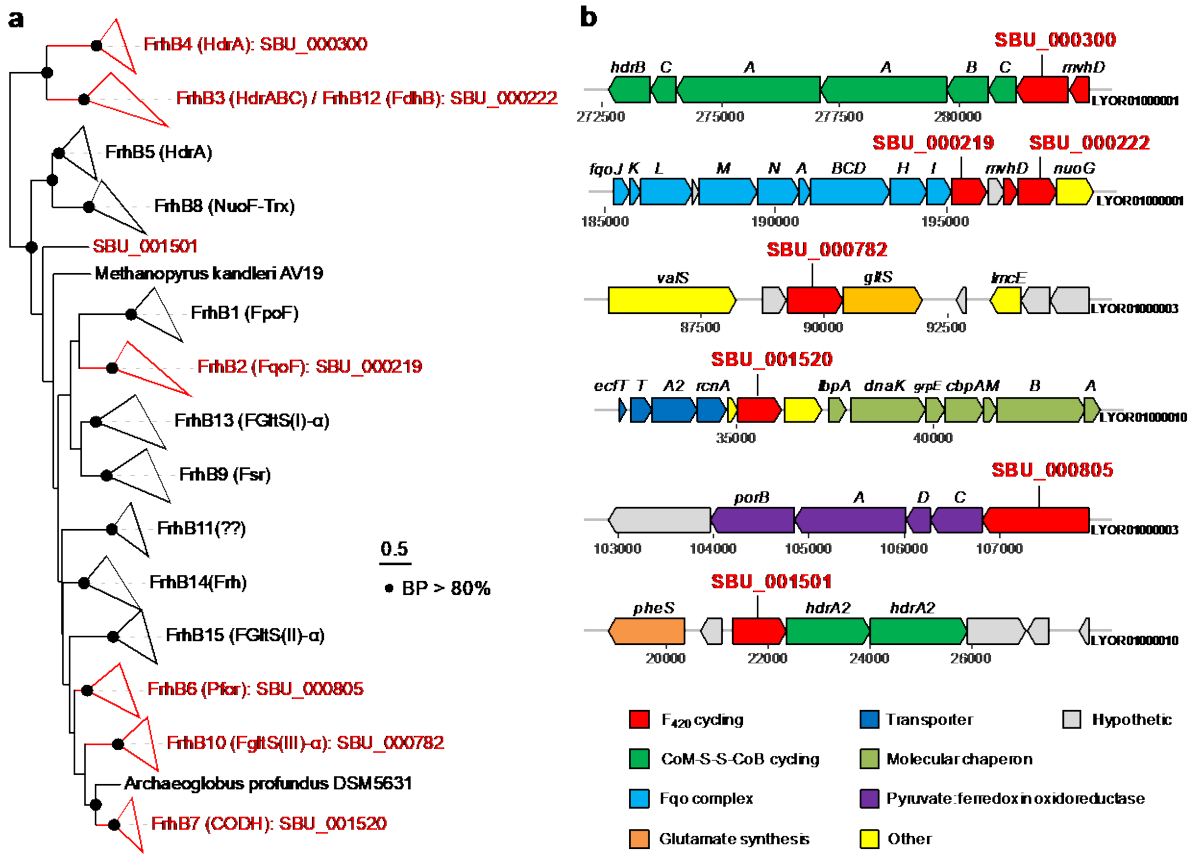

Supplementary Figure 10 | Phylogeny and genomic context of FrhB/FdhB homologs from *Ca*. S. butanivorans genome. **(a)** Maximum likelihood tree of FrhB/FdhB protein sequences. Homologs of FrhB/FdhB were retrieved from the genomes of *Ca*. S. butanivorans, ANME and methanogenic archaea. The major groups of the FrhB/FdhB protein family were collapsed, with the nomenclature for each clade as formerly defined[9]. The clades containing *Ca*. S. butanivorans FrhB/FdhB are highlighted in red. Tree scales represent substitutions per site. The branches with bootstrap values > 80% are shown with black circles; **(b)** Gene clusters harboring homologs of FrhB/FdhB in the *Ca*. S. butanivorans genome. Genes are shown as arrows. The gene names and/or locus tag are given above the gene arrows. The genomic coordinates (in base pair) and the contig id of each gene cluster are indicated below the line.

Supplementary Table 1

Oligonucleotide probes, helpers, and competitors specific for *Ca*. S. butanivorans and *Ca*. S. caldarius. The formamide concentration applied in CARD-FISH assays is shown. The table includes other oligonucleotide probes used in this study.

Probe name	Probe sequence	FA (%)	Target	Helpers and competitors	Reference
pSBU699	5'-GTCCCCCAGGTATCACAG-3'	30	*Ca*. S. butanivorans	hSBU680<br>5'-ATTTCACTCCTACTCCTG-3'<br><br>hSBU718<br>5'-GACGCCTTCGCCACAGGT-3'<br><br>Competitor<br>5'-GTCCCCCAGGGATCACAG-3'	This work
pSCAL871	5'-CCCCAAGTGGTGAGCTTC-3'	30	*Ca*. S. caldarius	hSCAL852<br>5'-CGGCTTCCCTACGGCACC-3'<br><br>hSCAL890<br>5'-TCAGCCTTGCGGCCGTAC-3'	This work
ARCH915	5'-GTGCTCCCCCGCCAATTCCT-3'	35	*Ca*. S. caldarius<br>*Ca*. S. butanivorans		[10]
EUB338I-III	5'-GCTGCCTCCCGTAGGAGT-3'<br>5'-GCAGCCACCCGTAGGTGT-3'<br>5'-GCTGCCACCCGTAGGTGT-3'	35	*Ca*. Desulfofervidus auxilii HotSeep1, other Bacteria		[11]<br>[12]
NON338	5'-ACTCCTACGGGAGGCAGC-3'	35	None		[13]

Supplementary Table 2

Additional gene features detected in *Ca*. S. butanivorans for refinement of the AOB pathway.

Gene	Feature	Locus tag
frhB/fdhB	coenzyme F420 hydrogenase subunit beta [EC:1.12.98.1]	SBU_000782
frhB/fdhB	coenzyme F420 hydrogenase subunit beta [EC:1.12.98.1]	SBU_000805
frhB/fdhB	coenzyme F420 hydrogenase subunit beta [EC:1.12.98.1]	SBU_001501
frhB/fdhB	coenzyme F420 hydrogenase subunit beta [EC:1.12.98.1]	SBU_001520
frhB/fdhB	coenzyme F420 hydrogenase subunit beta [EC:1.12.98.1]	SBU_000300
frhB/fdhB	coenzyme F420 hydrogenase subunit beta [EC:1.12.98.1]	SBU_000222
mqnA	chorismate dehydratase [EC:4.2.1.151]	SBU_000114
mqnB	futalosine hydrolase [EC:3.2.2.26]	SBU_000247
mqnC	cyclic dehypoxanthinyl futalosine synthase [EC:1.21.98.1]	SBU_000113
mqnD	1,4-dihydroxy-6-naphthoate synthase [EC:1.14.-.-]	SBU_000819
mqnE	aminodeoxyfutalosine synthase [EC:2.5.1.120]	SBU_000115
mqnX	aminodeoxyfutalosine deaminase [EC:3.5.4.40]	SBU_000112
NDH	NADH:quinone reductase (non-electrogenic) [EC:1.6.5.9]	SBU_001108
ACS	Acyl-CoA synthetase	SBU_000285
porB	Pyruvate:ferredoxin oxidoreductase, beta subunit	SBU_000801
porA	Pyruvate:ferredoxin oxidoreductase, alpha subunit	SBU_000802
porD	Pyruvate:ferredoxin oxidoreductase, delta subunit	SBU_000803
porC	Pyruvate:ferredoxin oxidoreductase, gamma subunit	SBU_000804

Supplementary Table 3

Stoichiometry of butane oxidation under different CO_2 labeling ratios. Anaerobic consumption of butane, and sulfate reduction to sulfide in the Butane50 enrichment culture (Bu50) were quantified in incubation experiments with different concentrations of ^{13}C in the CO_2/bicarbonate pool.

Experimental conditions, including label content [a]	C_4H_{10} added (mmol)	C_4H_{10} consumed (mmol)	Electrons in C_4H_{10} consumed (mmol) [b]	Sulfate reduced (mmol)	Electrons for sulfate reduction (mmol) [c]	Electron balance [d]
Bu50 + butane + 1.12% $^{13}CO_2$	0.41	0.40	8.45	0.99	7.92	1.07
Bu50 + butane + 25% $^{13}CO_2$	0.38	0.38	7.99	1.02	8.16	0.98
Bu50 + butane + 50% $^{13}CO_2$	0.39	0.39	8.07	0.94	7.52	1.07
Bu50 + butane + 75% $^{13}CO_2$	0.42	0.41	8.58	0.96	7.68	1.12
Bu50 + butane + 100% $^{13}CO_2$	0.39	0.39	8.26	1.06	8.48	0.97

[a] Experiments without addition of label contained natural abundance ^{13}C TIC; since the source ^{13}C-bicarbonate contained 98% ^{13}C, experiments with 25%, 50%, 75% and 100% label resulted in 25.3%, 49.6%, 73.8%, and 98% effective ^{13}C abundance, respectively.

[b] Calculated considering complete oxidation of butane ($C_4H_{10} + 12\ H_2O \rightarrow 4\ HCO_3^- + 30\ H^+ + 26\ e^-$), the amount of butane removed in the sterile control experiment, and an estimated 3% of butane removed through sampling.

[c] Calculated considering reduction of SO_4^{2-} to HS^- ($SO_4^{2-} + 8e^- + 9H^+ \rightarrow HS^- + 4\ H_2O$) and the amount of sulfate reduced in the control culture without addition of butane.

[d] Electrons available for dissimilation divided by electrons consumed by sulfate reduction. The average electron balance, 1.04, indicates that about 4% of the consumed butane was assimilated into biomass, comparable with the low methane assimilation rates of AOM consortia [14].

Supplementary Table 4

Standard free energy change associated with anaerobic oxidation of gaseous alkanes under sulfate reducing conditions [15]. The oxidation reaction become more exergonic with increasing alkane chain length. $\Delta G^{0\prime}$ calculations were done considering pH = 7.

Reaction	$\Delta G_{alkane}^{0\prime}$ (kJ mol^{-1}) [a]	$\Delta G_{sulfate}^{0\prime}$ (kJ mol^{-1}) [b]
CH_4 (g) + $SO_4^{2-} \rightarrow HCO_3^- + HS^- + H_2O$	-16.6	-16.6
$4C_2H_6$ (g) + $7SO_4^{2-} \rightarrow 8HCO_3^- + 7HS^- + 4H_2O + H^+$	-63.8	-36.5
$2C_3H_8$ (g) + $5SO_4^{2-} \rightarrow 6HCO_3^- + 5HS^- + 2H_2O + H^+$	-102.4	-41.0
$4C_4H_{10}$ (g) + $13SO_4^{2-} \rightarrow 16HCO_3^- + 13HS^- + 4H_2O + 3H^+$	-138.2	-42.5

[a]. $\Delta G_{alkane}^{0\prime}$: standard free energy per mol alkane

[b]. $\Delta G_{sulfate}^{0\prime}$: standard free energy per mol sulfate

Supplementary Table 5

Standard Gibbs energy of formation (ΔG_f^0) and activity coefficients (γ) for AOB reactants and products. Data was collected from Dean (1992)[16], Widdel and Musat (2019)[15], Wagman et al. (1968) [17], and Thauer et al. (1977) [8].

Compound	ΔG_f^0 (kJ mol^{-1})	ΔH_f^0 (kJ mol^{-1})	γ
C_4H_{10} (g)	-17.2	-74.9	1
SO_4^{2-} (aq)	-744.6	-909.3	0.11
HCO_3^- (aq)	-586.8	-689.93	0.55
HS^- (aq)	12.05	-20.62	0.55
H_2O (lq)	-237.18	-285.83	1
H^+ (aq)	0	0	1
H^+ (aq); pH = 7	-39.97	0	1

Supplementary Table 6

Description of terms, fluxes, rates, volumes, and constants.

Symbol	Description	Units
Amount of reaction substances and ^{13}C in each pool		
$[Alk_n]$	Amount of *n*-alkane (C_nH_{2n+2})	mmol
$[CO_2]$	Amount of CO_2 (including gaseous CO_2, aqueous CO_2, HCO_3^-, and CO_3^{2-})	mmol
$[^{13}C_{Alk_n}]$	Amount of ^{13}C atom in the *n*-alkane pool (C_nH_{2n+2})	mmol
$[^{13}C_{CO_2}]$	Amount of ^{13}C atom in the CO_2 pool	mmol
$[Alk_n]_0$[1]	Initial amount of *n*-alkanes (C_nH_{2n+2})	mmol
$[CO_2]_0$	Initial amount of CO_2 (including gaseous CO_2, aqueous CO_2, HCO_3^-, and CO_3^{2-})	mmol
$[C]_0$	Total amount of carbon in the system	mmol
$[^{13}C_{Alk_n}]_0$	Initial amount of ^{13}C atom in the *n*-alkane (C_nH_{2n+2}) pool	mmol
$[^{13}C_{CO_2}]_0$	Initial amount of ^{13}C atom in the CO_2 pool	mmol
$[^{13}C]_0$	Total amount of ^{13}C in the system	mmol
Forward flux, backward flux, and net flux		
f_+	Flux of substrate (*n*-alkane) arriving in product (CO_2) pool	mmol day^{-1}
f_-	Flux of product (CO_2) arriving in substrate (*n*-alkane) pool	mmol day^{-1}
v	Net flux of *n*-alkane oxidation process (measured as mmol butane time^{-1})	mmol day^{-1}
μ	Back flux extent (f_-/v)	/
Isotope ratio of carbon, ^{13}C abundance, and fractionation factors		
$^{13}R_{Alk_n}$	Isotope ratio of carbon (i.e., $^{13}C/^{12}C$) in *n*-alkane pool	/
$^{13}R_{co_2}$	Isotope ratio of carbon (i.e., $^{13}C/^{12}C$) in CO_2 pool	/
$^{13}R_+$	Isotope ratio of carbon in forward flux	/
$^{13}R_-$	Isotope ratio of carbon in backward flux	/
$\delta^{13}C_{Alk_n}$	$\delta^{13}C$ of *n*-alkane pool	/
$\delta^{13}C_{co_2}$	$\delta^{13}C$ of CO_2 pool	/
$^{13}F_{Alk_n}$	^{13}C abundance [$^{13}C/(^{12}C + ^{13}C)$] in *n*-alkane pool	/
$^{13}F_{co_2}$	^{13}C abundance [$^{13}C/(^{12}C + ^{13}C)$] in CO_2 pool	/
$^{13}F_+$	^{13}C abundance in forward flux	/
$^{13}F_-$	^{13}C abundance in backward flux	/
α_+	Fractionation factor of forward flux (butane oxidation)	/
α_-	Fractionation factor of backward flux (CO_2 fixation)	/
Volumes of the experimental system		
V_l	Volume of headspace (gas) in all experiments	ml
V_g	Volume of culture media (liquid) in all experiments	ml
V_T	Total experimental volume (gas + liquid)	ml
Constant		
R	gas constant	ml bar K^{-1} mmol^{-1}
T	Temperature	K
Reference value		
n	number of carbon atoms in *n*-alkane	/
$^{13}R_{\mathrm{Std}}$	$^{13}C:^{12}C$ ratio for the standard material VPDB	/
Parameters in first-order reaction model ($[Alk_n] = c_0 + (c_1 - c_0)\exp(-\lambda t)$)		
c_0	Lower asymptote of the model reaches when *t* goes infinity	mmol
c_1	Upper asymptote of the model attained at *t* = 0	mmol
λ	Rate constant	day^{-1}
Parameters in logistic model ($\delta\, ^{13}C_{Alk_n} = d_0 + (d_1 - d_0)/(1 + \exp(-k(t - d_2)))$)		
d_0	Lower asymptote of the model	/
d_1	Upper asymptote of the model	/
d_2	Time when half of the upper asymptote is reached	/
k	Logistic growth rate of $\delta^{13}C$ in *n*-alkane pool	day^{-1}
Parameters in likelihood Function used for Bayesian inference		
L	The likelihood function	
y	The experimentally measured data	
y_{model}	Predicted value from the model	
σ^2	The variance of error term	

[1] $[Alk_n] = \frac{cV_m}{24.77\ ml\ mol^{-1}}$

where n-alkane concentration (c; vol %) in headspace was measured using GC-FID; 24.77 ml mmol^{-1} stands for a conversion factor between moles and gas volumes, meaning 1 mmol of ideal gas occupies 24.77 ml at 298 K and 1 atmospheric pressure.

Supplementary Table 7

Parameters of exponential decay model (eq. 9) estimated from butane data with or without correction for butane loss due to sampling (<3%) or due to assimilation into biomass (4%). The model was applied to *n* = 5 replicates in the main back flux assays, with varied ^{13}C labelling of bicarbonate (1.12%, 25%, 50%, 75% and 98%; Figure 3A). Each parameter was shown as mean ± standard deviation. Two-sided T-test was applied to compare the estimated model parameters.

Parameters[1]	uncorrected	corrected	T test (P value; two-sided)[2]
c0	-0.00080 ± 0.0015	-0.00098 ± 0.0010	n.s., 0.831
c1	0.41 ± 0.011	0.42 ± 0.024	n.s., 0.607
λ	0.025 ± 0.020	0.024 ± 0.0019	n.s., 0.669

[1] parameters are from eq. 9
[2] n.s., not significant

Supplementary Table 8

Prior distribution for parameters in first-order reaction model and logistic model.

Model	Parameter	Prior
First-order reaction model	c_0	U(0,1)
	c_1	U(0,1)
	λ	U(0,0.2)
Logistic model	d_0	U(-200,200)
	d_1	U(-200,200)
	d_2	U(0,200)
	k	U(0,0.2)

U(l, u): uniform distribution with lower limit of l and upper limit of u.

Supplementary references

1 Laso-Pérez, R. *et al.* Thermophilic archaea activate butane via alkyl-coenzyme M formation. *Nature* **539**, 396-401 (2016). https://doi.org:10.1038/nature20152

2 Krukenberg, V. *et al.* Candidatus Desulfofervidus auxilii, a hydrogenotrophic sulfate-reducing bacterium involved in the thermophilic anaerobic oxidation of methane. *Environ. Microbiol.* **18**, 3073-3091 (2016). https://doi.org:10.1111/1462-2920.13283

3 Zeebe, R. E. & Wolf-Gladrow, D. *CO_2 in seawater: Equilibrium, kinetics, isotopes*. (Elsevier, 2001).

4 Dickson, A. G. & Goyet, C. Handbook of methods for the analysis of the various parameters of the carbon dioxide system in sea water. Version 2. Medium: ED; Size: 197 p. (United States, 1994).

5 Elsner, M., McKelvie, J., Lacrampe Couloume, G. & Sherwood Lollar, B. Insight into Methyl tert-Butyl Ether (MTBE) Stable Isotope Fractionation from Abiotic Reference Experiments. *Environ. Sci. Technol.* **41**, 5693–5700 (2007). https://doi.org:10.1021/es070531o

6 Jankowski, M. D., Henry, C. S., Broadbelt, L. J. & Hatzimanikatis, V. Group contribution method for thermodynamic analysis of complex metabolic networks. *Biophys. J.* **95**, 1487-1499 (2008). https://doi.org:10.1529/biophysj.107.124784

7 Thauer, R. K. Anaerobic oxidation of methane with sulfate: On the reversibility of the reactions that are catalyzed by enzymes also involved in methanogenesis from CO2. *Curr. Opin. Microbiol.* **14**, 292-299 (2011). https://doi.org:10.1016/j.mib.2011.03.003

8 Thauer, R. K., Jungermann, K. & Decker, K. Energy conservation in chemotrophic anaerobic bacteria. *Bacteriological Reviews* **41**, 100-180 (1977).

9 Chadwick, G. L. *et al.* Comparative genomics reveals electron transfer and syntrophic mechanisms differentiating methanotrophic and methanogenic archaea. *PLoS Biol.* **20**, e3001508 (2022). https://doi.org:10.1371/journal.pbio.3001508

10 Alm, E. W., Oerther, D. B., Larsen, N., Stahl, D. A. & Raskin, L. The oligonucleotide probe database. *Appl. Environ. Microbiol.* **62**, 3557-3559 (1996). https://doi.org:doi:10.1128/aem.62.10.3557-3559.1996

11 Amann, R. I., Krumholz, L. & Stahl, D. A. Fluorescent-oligonucleotide probing of whole cells for determinative, phylogenetic, and environmental studies in microbiology. *J. Bacteriol.* **172**, 762-770 (1990).

12 Daims, H., Brühl, A., Amann, R., Schleifer, K.-H. & Wagner, M. The Domain-specific Probe EUB338 is Insufficient for the Detection of all Bacteria: Development and Evaluation of a more Comprehensive Probe Set. *Syst. Appl. Microbiol.* **22**, 434-444 (1999). https://doi.org:https://doi.org/10.1016/S0723-2020(99)80053-8

13 Wallner, G., Amann, R. & Beisker, W. Optimizing fluorescent in situ hybridization with rRNA-targeted oligonucleotide probes for flow cytometric identification of microorganisms. *Cytometry* **14**, 136-143 (1993). https://doi.org:https://doi.org/10.1002/cyto.990140205

14 Nauhaus, K., Albrecht, M., Elvert, M., Boetius, A. & Widdel, F. In vitro cell growth of marine archaeal-bacterial consortia during anaerobic oxidation of methane with sulfate. *Environ. Microbiol.* **9**, 187-196 (2007). https://doi.org:10.1111/j.1462-2920.2006.01127.x

15 Widdel, F. & Musat, F. in *Aerobic Utilization of Hydrocarbons, Oils, and Lipids* (ed Fernando Rojo) Ch. Chapter 2, 33-72 (Springer International Publishing, 2019).

16 Dean, J. A. *Lange's handbook of chemistry*. (McGraw-Hill, 1992).

17 Wagman, D. D., Evans, W. H., Parker, V. B., Halow, I. & Bailey, S. M. *Selected values of chemical thermody- namic properties. Tables for the first thirty- four elements*. (U.S. Department ofCommerce, National Bureau of Standards, 1968).
