# Supplementary material for: Back flux during anaerobic oxidation of butane supports archaea-mediated alkanogenesis
Source: Nat Commun. 2024 Nov 7;15:9628. doi: 10.1038/s41467-024-53932-9 (PMC11543930; doi:10.1038/s41467-024-53932-9)
Supplement: Supplementary file 2 — Description of Additional Supplementary Files [file 41467_2024_53932_MOESM2_ESM.docx]

File Name: Supplementary Data 1

Description: Genomes of anaerobic multicarbon alkane-oxidizing archaea (ANKA) and anaerobic methanotrophic (ANME) archaea included in comparative genomics.

File Name: Supplementary Data 2

Description: Syntropharchaeia genomes included in comparative genomics.

File Name: Supplementary Data 3

Description: Protein families that are overrepresented in multicarbon alkane-oxidizing archaea (ANKA) genomes versus anaerobic methanotrophic archaea (ANME).

File Name: Supplementary Data 4

Description: Protein families that are overrepresented in ACR-encoding Syntropharchaeia (ACR_Syn) versus MCR-encoding Syntropharchaeia (MCR_Syn).

File Name: Supplementary Data 5

Description: AcrA sequences retrieved from Musat et al. 2024 (<https://doi.org/10.1016/j.mib.2024.102486>).

File Name: Supplementary Data 6

Description: Derivation of ΔG^0^' for each metabolic step of AOB.

File Name: Supplementary Data 7

Description: Derivation of ΔG^0^' for each metabolic step of rAOB.

File Name: Supplementary Data 8

Description: Presence and relative abundance of ACR-encoding genera across biomes.

File Name: Supplementary Data 9

Description: AcrA protein fragments retrieved from IMG/M database. All fragments branches with divergent AcrA sequences based on RAxML EPA analysis.
