# Supplementary material for: Back flux during anaerobic oxidation of butane supports archaea-mediated alkanogenesis
Source: Nat Commun. 2024 Nov 7;15:9628. doi: 10.1038/s41467-024-53932-9 (PMC11543930; doi:10.1038/s41467-024-53932-9)
Supplement: Supplementary file 4 — Reporting Summary [file 41467_2024_53932_MOESM4_ESM.pdf]

nature portfolio

Corresponding author(s): Florin Musat

Last updated by author(s): Oct 8, 2024

Reporting Summary

Nature Portfolio wishes to improve the reproducibility of the work that we publish. This form provides structure for consistency and transparency in reporting. For further information on Nature Portfolio policies, see our Editorial Policies and the Editorial Policy Checklist.

Statistics

For all statistical analyses, confirm that the following items are present in the figure legend, table legend, main text, or Methods section.

n/a	Confirmed	
☐	☒	The exact sample size (*n*) for each experimental group/condition, given as a discrete number and unit of measurement
☒	☐	A statement on whether measurements were taken from distinct samples or whether the same sample was measured repeatedly
☐	☒	The statistical test(s) used AND whether they are one- or two-sided<br>*Only common tests should be described solely by name; describe more complex techniques in the Methods section.*
☒	☐	A description of all covariates tested
☒	☐	A description of any assumptions or corrections, such as tests of normality and adjustment for multiple comparisons
☐	☒	A full description of the statistical parameters including central tendency (e.g. means) or other basic estimates (e.g. regression coefficient) AND variation (e.g. standard deviation) or associated estimates of uncertainty (e.g. confidence intervals)
☐	☒	For null hypothesis testing, the test statistic (e.g. *F*, *t*, *r*) with confidence intervals, effect sizes, degrees of freedom and *P* value noted<br>*Give P values as exact values whenever suitable.*
☐	☒	For Bayesian analysis, information on the choice of priors and Markov chain Monte Carlo settings
☒	☐	For hierarchical and complex designs, identification of the appropriate level for tests and full reporting of outcomes
☒	☐	Estimates of effect sizes (e.g. Cohen's *d*, Pearson's *r*), indicating how they were calculated

Our web collection on statistics for biologists contains articles on many of the points above.

Software and code

Policy information about availability of computer code

Data collection	Imaging: Zeiss ZEN Pro 3.1, ZEN Blue 3.8<br>GC-IRMS: Thermo Fisher Scientific Isodat v. 3.0
Data analysis	The data in this study was analyzed and visualized using R (v3.6.3) and Python (v3.9). Custom code was used for thermodynamic modelling and to interpret the back flux results. The code is submitted and publicly available at GitHub and Zenodo (https://zenodo.org/records/13895334).<br>Other software used for data analysis: SILVA database release 111; ARB v 6.0.6; R v. 4.4.1; cutadapt v. 0.2.2; R package DADA2 v. 1.28.0; SILVA reference database 138; R package Decontam v. 1.20.0; CheckM 1.2.2; prodigal v2.6.3; diamond v0.9.24.125; Psortb v3.0; HMMER 3.2.1; mafft (v7.407); RAxML v 7.2.6; iTOL; GTDB database r220

For manuscripts utilizing custom algorithms or software that are central to the research but not yet described in published literature, software must be made available to editors and reviewers. We strongly encourage code deposition in a community repository (e.g. GitHub). See the Nature Portfolio guidelines for submitting code & software for further information.

Data

Policy information about availability of data

All manuscripts must include a data availability statement. This statement should provide the following information, where applicable:

- Accession codes, unique identifiers, or web links for publicly available datasets
- A description of any restrictions on data availability
- For clinical datasets or third party data, please ensure that the statement adheres to our policy

The bioinformatics, thermodynamic modelling, and isotope data generated in this study are provided in the Supplementary Information. The sequencing data are available in the NCBI database under the accession code BioProject ID PRJNA1134092 [https://www.ncbi.nlm.nih.gov/bioproject/PRJNA1134092/]. Source data are provided with this paper.

Research involving human participants, their data, or biological material

Policy information about studies with human participants or human data. See also policy information about sex, gender (identity/presentation), and sexual orientation and race, ethnicity and racism.

Reporting on sex and gender	This study does not involve human participants.
Reporting on race, ethnicity, or other socially relevant groupings	This study does not involve human participants.
Population characteristics	This study does not involve human participants.
Recruitment	This study does not involve human participants.
Ethics oversight	Not applicable

Note that full information on the approval of the study protocol must also be provided in the manuscript.

Field-specific reporting

Please select the one below that is the best fit for your research. If you are not sure, read the appropriate sections before making your selection.

☒ Life sciences ☐ Behavioural & social sciences ☐ Ecological, evolutionary & environmental sciences

For a reference copy of the document with all sections, see nature.com/documents/nr-reporting-summary-flat.pdf

Life sciences study design

All studies must disclose on these points even when the disclosure is negative.

Sample size	No statistical methods were used to predetermine sample size
Data exclusions	No data were excluded from the analysis
Replication	Microbial community of the Butane50 culture, microscopy, imaging: native aggregates, n = 3; cell counting and abundance, n = 3 independent cultures, n > 60 images per test condition, n > 5000 cells counted in CARD-FISH assays<br>Main back flux assay (6 mM butane and 28 mM sulfate) was done with n = 9 biological replicates as follows: natural abundance 13C-bicarbonate, n = 3; 25%, 50% and 75% 13C-bicarbonate, n = 1; 98% 13C-bicarbonate n = 3. Controls without butane, n = 4. Back flux assays with limiting sulfate concentration (5 mM), n = 3. Back flux assays with different starting butane concentrations, n = 3. For all back flux assays and time points sampled, butane concentrations were measured with n = 2 replicates, and isotope analyses with n = 3 replicates.
Randomization	The experiments were not randomized, since all analyses concerned a single enrichment culture
Blinding	The investigators were not blinded to allocation during experiments and outcome assessment

Reporting for specific materials, systems and methods

We require information from authors about some types of materials, experimental systems and methods used in many studies. Here, indicate whether each material, system or method listed is relevant to your study. If you are not sure if a list item applies to your research, read the appropriate section before selecting a response.

Materials & experimental systems

n/a	Involved in the study
☒	☐ Antibodies
☒	☐ Eukaryotic cell lines
☒	☐ Palaeontology and archaeology
☒	☐ Animals and other organisms
☒	☐ Clinical data
☒	☐ Dual use research of concern
☒	☐ Plants

Methods

n/a	Involved in the study
☒	☐ ChIP-seq
☒	☐ Flow cytometry
☒	☐ MRI-based neuroimaging

Plants

Seed stocks	*Report on the source of all seed stocks or other plant material used. If applicable, state the seed stock centre and catalogue number. If plant specimens were collected from the field, describe the collection location, date and sampling procedures.*
Novel plant genotypes	*Describe the methods by which all novel plant genotypes were produced. This includes those generated by transgenic approaches, gene editing, chemical/radiation-based mutagenesis and hybridization. For transgenic lines, describe the transformation method, the number of independent lines analyzed and the generation upon which experiments were performed. For gene-edited lines, describe the editor used, the endogenous sequence targeted for editing, the targeting guide RNA sequence (if applicable) and how the editor was applied.*
Authentication	*Describe any authentication procedures for each seed stock used or novel genotype generated. Describe any experiments used to assess the effect of a mutation and, where applicable, how potential secondary effects (e.g. second site T-DNA insertions, mosiacism, off-target gene editing) were examined.*
